# Characterization of Total Phenolic Constituents from the Stems of *Spatholobus suberectus* Using LC-DAD-MS^n^ and Their Inhibitory Effect on Human Neutrophil Elastase Activity

**DOI:** 10.3390/molecules18077549

**Published:** 2013-06-27

**Authors:** Youwu Huang, Liang Chen, Li Feng, Fujiang Guo, Yiming Li

**Affiliations:** School of Pharmacy, Shanghai University of Traditional Chinese Medicine, Shanghai 201203, China

**Keywords:** *Spatholobus suberectus*, phenolic components, HPLC-DAD/ESI-MS, procyanidins, human neutrophil elastase

## Abstract

*Spatholobus suberectus* Dunn, belonging to the legume family (Fabaceae), has been used as a Traditional Chinese Medicine for the treatment of anemia, menoxenia and rheumatism. A limited number of studies report that various types of flavonoids are the main characteristic constituents of this herb. We have now found that *S. suberectus* contains about 2% phenolic components and characterized the major phenolic components as homogeneous B-type procyanidin conjugates using a liquid chromatography with diode-array detection-ESI mass spectrometry (LC-DAD/ESI-MS) method. This is the first report on occurrence of most B-type procyanidins in this herb. Moreover, the total phenolics extract was assayed for inhibitory activity on human neutrophil elastase and its IC_50_ was found to be 1.33 μg/mL.

## 1. Introduction

*Spatholobus suberectus* Dunn, belonging to the legume family (Fabaceae), is called “ji xue teng” in China because it produces a chicken blood-like juice when its bark is broken [1]. The stem of *S. suberectus* has been used as a Traditional Chinese Medicine for the treatment of anemia, menoxenia and rheumatism [2,3]. A limited number of studies have been conducted that report various types of flavonoids are the principal characteristic components in this herb [4], including flavone, isoflavones, flavanones, flavanonols, and chalcone [3,5,6]. Other chemical investigations have led to isolation of several phenolic glycosides, quinones [1], steroids, some fatty acids and two procyanidins [7]. An isoflavone *i.e.*, formononetin, is used now in the Chinese Pharmacopoeia as a qualitative marker compound for identification of *S. suberectus*, but it is hard to provide a comprehensive reference in order to systematically evaluate the quality of *S. suberectus* by only marking an isoflavone. One study also provided a reference using six index flavonoids in order to systematically evaluate the quality of *S. suberectus* by a HPLC method [8]. Here, we report the identification of some major B-type procyanidin components by HPLC-DAD-MS^n^. This is the first report on occurrence of most B-type procyanidins in this herb.

Moreover, the total phenolics extract was assayed for inhibitory activity on human neutrophil elastase (HNE) (EC 3.4.21.37). HNE, a member of the chymotrypsin family of serine proteases, is primarily stored in the azurophil granules of neutrophil granulocytes. HNE can degrade a wide variety of biomacromolecules that exhibit important biological functions, especially in the degradation of a wide range of extracellular matrix proteins, including fibronectin, laminin, proteoglycans, collagens, and elastin [9]. There is little argument that tissues exposed to uninhibited HNE will suffer damage. It will play a destructive role as a deficiency of the endogenous inhibitor of HNE [10]. Insufficient levels of protease inhibitors have been suggested as a contributing factor in a number of diseases including acute lung injury, cystic fibrosis, ischemic reperfusion injury, rheumatoid arthritis, atherosclerosis, psoriasis, and malignant tumors [11]. Sivelestat, which is currently used as a synthetic elastase inhibitor, is applied for the treatment of acute lung injury and acute respiratory distress syndrome (ARDS). However, sivelestat did not improve mortality outcome in the treatment [12]. A number of reports about natural HNE inhibitors have also been published [13,14,15,16]. During our HNE inhibition screening program, the total phenolics extract showed remarkable inhibitory effects and its IC_50_ was found to be 1.33 μg/mL.

## 2. Results and Discussion

### 2.1. Identification of Phenolic Constituents

**Figure 1:**
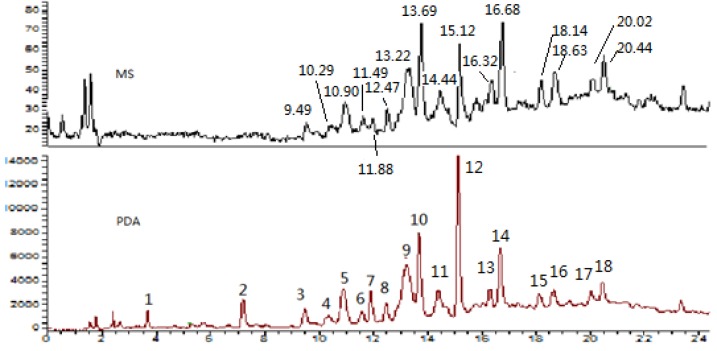
HPLC-DAD-ESI-MS profile of the ethyl acetate fraction of *S. suberectus* MS: (+) ESI; PDA: (280 nm).

**Figure 2:**
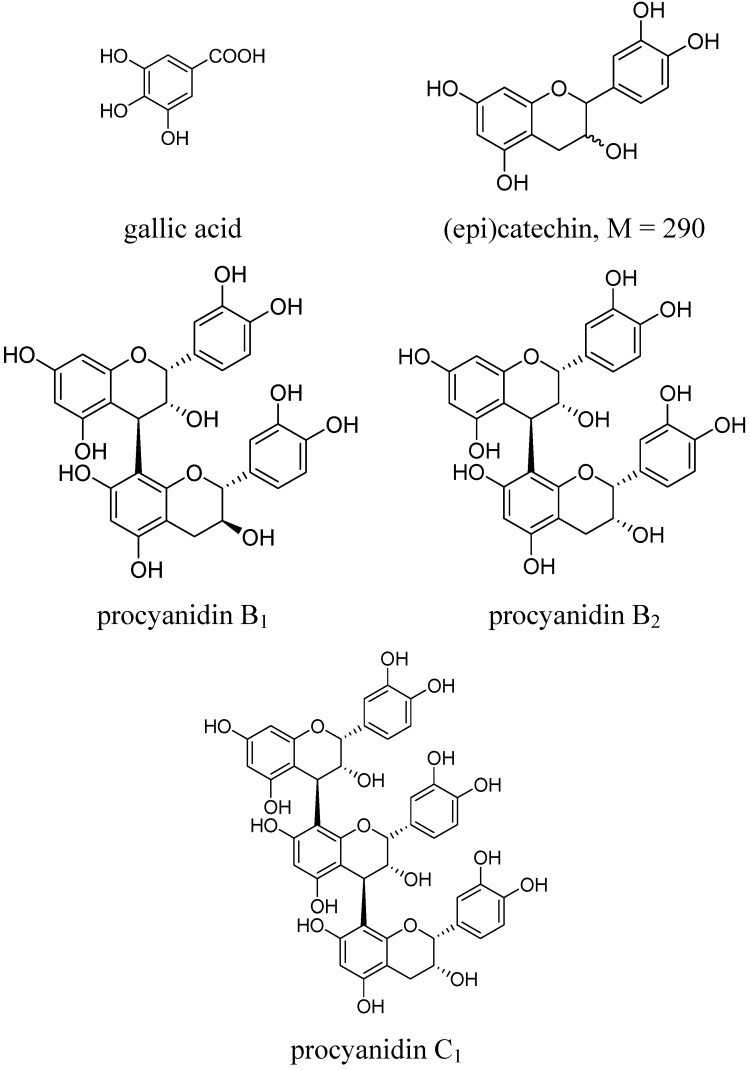
Chemical structures of the identified compounds in extract of *Spatholobus suberectus.*

The five batches of stems of *S. suberectus* were collected from different herbal medicine markets in China. The analysis of all raw materials showed similar DAD or TIC profiles. The total ion current chromatogram obtained from the LC/MS analysis and HPLC-DAD profile at 280 nm are shown in Figure 1. A total of eighteen major peaks were detected in the HPLC chromatogram. Identification of these peaks is based on retention times, UV absorption maxima, and mass spectra or comparisons with authentic compounds. All results were summarized in Table 1. Peaks **3**–**18** exhibit absorbance maxima at two wavelengths (232 and 279 nm), suggesting they belong to the same class of phenolic compounds. One phenolic acid and two flavan-3-ols were unambiguously identified as gallic acid (peak **1**) and catechin (peak **7**) and epicatechin (peak **12**) by comparison with the authentic standards (Figure 2). Two procyanidin dimers B_1_ (peak **3**) and B_2_ (peak **10**) and a procyanidin trimer C_1_ (peak **13**) were also assigned by comparing them to the reference compounds isolated from *Cinnamomum cassia* in our lab (Figure 2). Peaks **5** and **9** both gave protonated species with *m/z* 579 in positive ion mode and deprotonated molecules at *m/z* 577 in negative ion mode, suggesting they were also B-type procyanidin dimers of (epi)catechin linked via C4-C6 or C4-C8 [17]. MS^2^ experiments were further employed to identify these procyanidins. In the MS^2^ spectra of these deprotonated procyanidins (*m/z* 577), important intense negative ions at *m/z* 425 (loss of 152 amu) are typically observed, which indicates a characteristic fragmentation pathway of a retro Diels-Alder reaction [18].

**Table 1:** Phenolic constituents identified or identified tentatively in *Spatholobus suberectus*.

**No.**	**tR (min)**	**UV (nm)**	**Ion (+)**	**Ion (-)**	**MS^2^(-)**	**Compounds**
1	3.68	214, 273				gallic acid
2	7.20	218,259,293	579			unknown
3	9.49	232, 279	579	577	425, 407, 289	procyandin B1
4	10.29	232, 278	595	593	425, 407, 305, 289	prodelphinidin dimer
5	10.90	232, 278	579	577	425, 407, 289	procyandin B-type dimer
6	11.49	233, 279	867	865	739, 695, 577, 425, 287	procyandin B-type trimer
7	11.97	229, 279		289	245, 205	catechin
8	12.47	228, 279	563, 867	561, 865	435, 289, 739, 695, 577, 425, 287	propelargonidin dimer and procyandin trimer
9	13.22	232, 278	579	577	425, 407, 289	procyandin B-type dimer
10	13.69	232, 278	579	577	425, 407, 289	procyandin B2
11	14.44	230, 279	867, 889	865	739, 695, 577, 425, 287	procyandin B-type trimer
12	15.12	232, 278	291	289	245, 205	epicatechin
13	16.32	232, 279	889, 867	865	739, 695, 577, 425, 287	procyanidin C1
14	16.68	232, 278	563, 585	561	543, 435, 289	propelargonidin dimer
15	18.14	232, 278	851, 873	849, 1137	697, 577, 559, 407, 289, 967, 847, 695, 577	propelargonidin trimer and tetramer
16	18.63	232, 278	851, 873, 1139	849, 1137	697, 577, 559, 407, 289, 967, 847, 695, 577	propelargonidin trimer and tetramer
17	20.02	233, 279	867, 1155	865, 1153	739, 695, 577, 425, 287 983, 865, 695, 577	procyanidin trimer and tetramer
18	20.44	232, 278	835, 1155	833, 1153	561, 543, 983, 865, 695, 577	propelargonidin trimer and procyanidin tetramer

**Figure 3:**
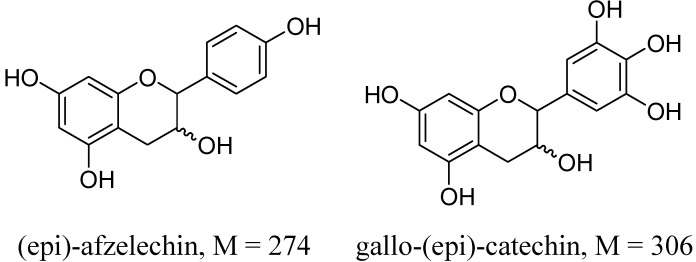
Chemical structures of compounds mentioned in paper.

The mass spectrum of the compound eluting at t_R_ 16.68 min (Peak **14**) exhibited a positive peak at *m/z* 563 (M+H)^+^ and a negative peak at *m/z* at 561, which implied it was a propelargonidin B-type dimer composed of one (epi)catechin and one (epi)afzelechin unit (Figure 3).

Loss of 272 amu in its MS^2^ originated from (epi)afzelechin. Peak **4** eluting at t_R_ 10.29 min with molecular weight 594 was assigned to be a prodelphinidin dimer of (epi)catechin and (epi)gallocatechin (305 amu) (Figure 3). ESI-MS of peaks **6** and **11** revealed [M+H]^+^ at *m/z* 867 and [M−H]^–^ at *m/z* at 865 and main fragmentation peaks at *m/z* 577 and 287 amu, suggestion they were procyanidin B-type trimers of three (epi)catechins. A peak (peak **8**) with molecular weights at 562 and 866 was attributed to a mixture containing a propelargonidin B-type dimer of one (epi)catechin with one (epi)afzelechin and a procyanidin B-type trimer of three (epi)catechins. Both peaks **15** and **16** were also a mixture of propelargonidin trimer [two (epi)catechins and an afzelechin] and tetramer [three (epi)catechins and an afzelechin] based on the molecular ions at 850 and 1,138. Peak **17** was concluded to be a mixture of procyanidin trimer [three (epi)catechins] and procyanidin tetramer [four (epi)catechins] based on the molecular weights at 866 and 1,154 with characteristic MS^2^ fragments [19]. Peak **18** was concluded to be a mixture of propelargonidin trimer [one (epi)catechin and two afzelechins] and a procyanidin tetramer [four (epi)catechins] based on the molecular weights of 834 and 1,154. Peak **2** could not be identified. It exhibited a positive peak at *m/z* 279 (M+H)^+^ and presented absorbance maxima at three wavelengths 221, 259 and 293 nm, which differ from those of procyanidins.

### 2.2. Total Phenolic Contents

Phenolic contents in the raw materials were investigated by a colorimetric method [20]. According to the test results, the five batches of raw materials contained between 1.81%–2.60% total phenolics.

### 2.3. Inhibition of HNE

The HNE inhibitory activities of five different concentrations (0.5, 1.5, 5, 10 and 50 μg/mL) of the test samples are listed in Table 2. The results showed the inhibitory effect of the phenolic extract of *S. suberectus* (containing 81.4% phenolic contents) on HNE increased along with concentration. As a result, total phenolics dose-dependently inhibited HNE activity at the level of IC_50_ 1.33 μg/mL.

**Table 2:** Inhibition of total phenolics on HNE activity.

**Sample**	**Blank**	**0.5 µg/mL**	**1.5 µg/mL**	**5 µg/mL**	**15 µg/mL**	**50 µg/mL**
Phenolics	0 ± 1.67%	40.97 ± 1.06%	53.26 ± 0.61%	60.09 ± 1.37%	63.73 ± 1.67%	66.46 ± 0.76%

Values are presented as mean ± SD.

## 3. Experimental

### 3.1. Plant Materials

Five batches of the stems of *S. suberectus* were collected in China from the different herbal medicine markets in 2012. Raw materials were authenticated by Prof. Zhili Zhao (the School of Pharmacy, Shanghai University of Traditional Chinese Medicine). Voucher specimens have been deposited in the Natural Products Chemistry Laboratory of the School of Pharmacy, Shanghai University of Traditional Chinese Medicine, China.

### 3.2. Preparation of Total Phenolics

Each dried sample (100 g) was ground and stored in a freezer. Powdered samples (10 g) were sonicated three times in a mixture of ethanol/water (50:50, v/v, 3 × 200 mL) for 30 minutes each time. The combined 50% EtOH extract was reduced in volume to 200 mL under vacuum and then extracted with CH_2_Cl_2_ (3 × 200 mL). The CH_2_Cl_2_ layer was discarded and the water layer was extracted with EtOAc (3 × 200 mL). The EtOAc layer was collected and evaporated to dryness. Finally, the samples were dissolved in methanol and water (50:50, V/V) and diluted to about 1 mg/mL. The samples were filtered and analyzed by HPLC-DAD-MS^n^.

### 3.3. LC-MS Apparatus

HPLC analysis was carried out on a Shimadzu LC-20ADXR HPLC system (Shimadzu, Kyoto, Japan) equipped with a SPD-M20A PDA detector. A 5 μm, C_18_, 150 mm×4.6 mm Syncronis column (Thermo Fisher Scientific Inc., Waltham, MA, USA) was used to separate the phenolic constituents. The chromatographic conditions were as follows: flow rate of 1.0 mL/min; solvent A, CH_3_CN; solvent B, 0.2% acetic acid; 0 min, 6:94 (A:B, v:v); 10 min, 13:87; 20 min, 23:77; 25 min, 45:55; 30 min, 80:20. MS analysis was performed on a Thermo LCQ Fleet mass ion-trap spectrometer (Thermo Fisher Scientific Inc.). Full-scan mass spectra were acquired in electro spray ionization (ESI) mode with collision energy of 35 eV, over the mass range *m/z* 150–2,000. The sheath gas and the auxiliary gas flow rate were 40 and 10 (arbitrary units) separately and the sweep gas flow rate was set at 0.03. The spray voltage was set at 4.5 kV and the capillary voltage was −37. The capillary temperature was set at 320 °C. 

### 3.4. Phenolic Content Assay

Phenolic contents in raw materials were assayed by a colorimetric method [20]. In this procedure, to prepare standard solutions, 1, 2, 3, 4, and 5 mL aliquots of 0.05 mg/mL of gallic acid solution were taken from and transferred into five 25 mL of volumetric flasks, then mixed separately with 1 mL of molybdic acid and 11, 10, 9, 8, and 7 mL of water. After that, the five volumetric flasks were made up to volume with 20% Na_2_CO_3_ solution. The blank and standard solutions were prepared using the same method, but 1 mL of water or 1 mL of extract solution (0.2 mg/mL) were separately used instead of gallic acid. The sample solution, blank and standard solutions were kept at room temperature for 30 min and the absorbance read at 760 nm. The mean of two readings was used as the result.

### 3.5. Inhibition of HNE

The HNE inhibitory activity of the test sample was evaluated using a previously described procedure [16] with sivelestat as positive control. Briefly, substrate solution (200 μL, 1.4 mM MeO-Suc-Ala-Ala-Pro-Val-pNA in Tris-HCl buffer, 10 mM, pH 7.5) was mixed with different concentration ranges of test solution (stock solutions of samples were dissolved in dimethyl sulfoxide and diluted with Tris-HCl buffer to give the final sample concentrations), enzyme solution (1 μL, 0.01 U HNE) was added and the mixture was incubated for 1 h at 37 °C in the dark. Then the reaction was quenched by adding soybean trypsin inhibitor (200 μL) at the concentration of 0.2 mg/mL, and the absorbance was immediately measured at 405 nm using a plate reader. 10 mM pH 7.5 Tris-HCl buffer was used as a zero alignment of the test samples. HNE inhibitory activity was calculated using the following equation:

HNE inhibitory activity = [A_control_ − (A_sample_ − A_zero_)]/A_control_ × 100%

where:

A_control_: the absorbance of the group with only HNE and the substrate.
A_sample_: the absorbance of the group with test samples, substrate and HNE.
A_zero_: the absorbance of the group with test samples, Tris-HCl buffer and HNE.

The relative activities of test samples were expressed as IC50 values.

## 4. Conclusions

In our study, over ten phenolic components from *S. suberectus* were characterized by comparison with reference compounds or tentatively identified using a liquid chromatograph with diode-array detection-ESI mass spectrometry (LC-DAD/ESI-MS) method. These major procyanidins could be identified as homogeneous B-type ones according to their structural features, and are composed of flavan-3-ol units linked through C-C bonds. No A-type procyanidin which coexist with C-O-C linkages were detected. This is the first report on the occurrence of most B-type procyanidins in this herb, although they are widely and generally abundant. In addition, the total phenolics extract showed effective inhibitory activity on human neutrophil elastase and its IC_50_ was found to be 1.33 μg/mL.
